# Effect of apparatus characteristics on anxiety-like behavior in young adult and old mice of both sexes assessed by the elevated plus maze assay

**DOI:** 10.3389/fnbeh.2023.1182661

**Published:** 2023-08-10

**Authors:** Lauren Gaspar, Sydney Bartman, Giuseppe Coppotelli, Jaime M. Ross

**Affiliations:** ^1^George and Anne Ryan Institute for Neuroscience, University of Rhode Island, Kingston, RI, United States; ^2^Department of Biomedical and Pharmaceutical Sciences, College of Pharmacy, University of Rhode Island, Kingston, RI, United States

**Keywords:** elevated plus maze, aging, sex differences, behavior, rodents

## Abstract

Incidence of anxiety-like disorders in humans has been shown to decrease with aging; however, it is still under debate whether there are similarities in mice, which would support the use of mouse models in understanding the neuronal network changes that regulate anxiety-like behavior in aging. One of the most common tests used to assess anxiety-like behavior in laboratory animals is the elevated plus maze (EPM). Although several variables, such as room brightness and width of the maze arms, have been shown to influence the spontaneous animal behavior during the EPM test, none of these variables have ever been evaluated in aging to understand their possible differential effect on younger and older mice. We therefore decided to investigate the effect of apparatus construction on young adult and old mice of both sexes on EPM test performance. Our results show that distance traveled during the test is the variable that is most affected by apparatus characteristics independent of age and sex. We also found that apparatus construction was key in demonstrating that old mice spent more time and had relatively more entries in the open arms as compared to young mice, suggesting a decrease in anxiety-like behavior with age. Taken together, our data demonstrate that EPM apparatus characteristics dramatically affect test outcome with a wider arm apparatus being more effective in revealing age-dependent changes in anxiety-like behavior, thus, suggesting the use of a wider arm EPM when conducting aging studies in mice.

## Introduction

**FIGURE 1:**
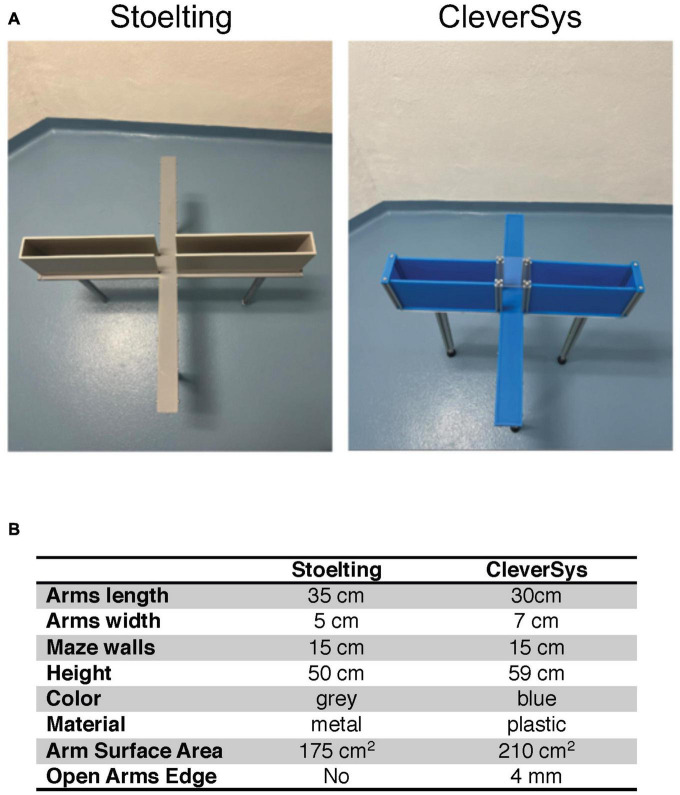
Elevated plus maze (EPM) apparatuses. **(A)** Image of the two apparatuses used in the study. **(B)** Summary table of the characteristics of the apparatuses.

One of the behavioral tests used to assess what is considered to be anxiety-like behavior in laboratory rodents is the elevated plus maze (EPM) (Carobrez and Bertoglio, 2005; Walf and Frye, 2007; Pawlak et al., 2008). Genetic and chemical effects on EPM performance in mice have been widely demonstrated and the EPM is presently the most used assay to test modulators of anxiety-like behaviors in pharmaceutical drug discovery settings (Bourin, 2015). Initial experiments using the EPM assay to assess anxiety-like behaviors were performed by Handley and Mithani (1984) in rats using an apparatus with 45 cm long by 10 cm wide arms and a total maze height of 70 cm. The assay was developed to detect the effect of anxiolytic and anti-anxiolytic drugs in rats and later was validated for mouse studies as well (Lister, 1987). The apparatus consists of two open and two enclosed arms, crossed perpendicularly to form a center platform with a “plus sign” shape raised about one meter above the floor (Komada et al., 2008; see Figure 1). The test exploits the conflict between the natural adversity of mice for open and elevated space and their natural curiosity to explore a novel environment (File, 1996). The test takes advantage of a phenomenon known as thigmotaxis, which is the tendency of mice to remain in physical contact with walls and explains why rodents avoid open spaces in their natural environment. Thigmotactic behavior is considered to result from anxiety and is affected by both anxiolytic and anxiogenic reference drugs, thus, may be used as a reliable measure of anxiety (Simon et al., 1994). Rodents who enter the open arms more frequently and spend more time in the open arms are thought to exhibit less anxiety-like behavior due to their willingness to explore the open space, as compared to mice that spend the majority of the time in the closed arms (Rodgers and Dalvi, 1997).

Using animal models of human diseases for the purpose of finding treatments is a common practice among scientists; however, when it comes to mimicking neurological disorders, the use of animal models poses many obvious limitations, since conditions such as depression and anxiety are only accessible in humans by self-report (Jucker, 2010; Chesselet and Carmichael, 2012). Rodents make up approximately 95% of all animals used in research with mice being the most utilized due to wide availability of genetic models (Hickman et al., 2017); however, it is still debated if changes in anxiety-like behaviors in mice during aging recapitulate human findings, justifying the use of mouse models to study how brain aging impacts this aspect of behavior (Byers et al., 2010; Goodwin et al., 2020; Darvas et al., 2021).

When the EPM has been used to test whether aging affects anxiety-like behaviors in mice, the results have not been consistent, with many studies reporting contradictory findings. For example, an investigation conducted with a large number of C57BL/6J mice up to 12 months-old showed that while the distance traveled in the open arms declines with aging, time spent and percentage entries in the open arm significantly increased, suggesting a decrease in anxiety-like behavior from young to middle age (Shoji et al., 2016), with the same conclusions found in rats (Bessa et al., 2005). A different study reported no change in the percentage of entries as well as the time spent in the open arms in old mice, suggesting that aging does not affect anxiety-like behavior as measured by the EPM test (Shoji and Miyakawa, 2019). On the other hand, Li et al. (2020) reported a decrease in time spent and percentage entries in the open arms during aging in mice, with similar results found by others (Bedrosian et al., 2011; Hoffman et al., 2017). Thus, there is a lack of consensus on how aging affects anxiety-like behavior in mice as assessed by the EPM test.

Several factors have been shown to affect the EPM test, including mouse strain, room illumination, and maze construction (Lamberty and Gower, 1996), with the latter not adequately considered in aging studies, which could explain the inconsistent results previously reported when testing old mice. Despite Lamberty and Gower (1996) clearly demonstrating more than twenty years ago that EPM arm width can affect performance in some mouse strains, there still is no consensus with regards to standard dimensions of the EPM apparatus for rat and mouse testing. Currently available EPM apparatuses are being used interchangeably yet they vary in dimensions, including height of the maze from the floor, length and width of the maze arms ranging from 5 to 10 cm, height of the enclosure of the enclosed arms, color and material used, as well as presence or absence of a small rim surrounding the edge of the open arms that can prevent mice from falling when exploring the open arms (Wiley et al., 1995; Hoffman et al., 2017; Bello-Arroyo et al., 2018; Yoshizaki et al., 2020; Heinz et al., 2021). The assumption that different models with minor variations are all reliable in detecting anxiety-like behaviors has contributed to a lack of consistency in maze construction and has resulted in companies marketing EPM apparatuses with different dimensions and specifications. A further element of confusion is that studies often report only one parameter to support their findings out of the several obtained from the EPM assay, such as duration, number of entries (bouts), percentage entries, and distance traveled in both the open and closed arms, which does not allow for complete understanding of the rodent’s performance and behavior exhibited in the assay.

To determine if variation in EPM characteristics, including arm width, material, and presence of a rim surrounding the edge of the open arms can affect performance, we tested ∼3 and 24 month-old mice of both sexes using two different apparatuses obtained from two vendors and compared how maze characteristics affect EPM test outcome, including the number and percentage of entries, time spent, and distance traveled in both the open and closed arms.

## Materials and methods

### Mice

Old female and male C57BL/6J mice were obtained from the National Institute of Aging (NIA) aged rodent colony (Charles River Laboratories, Kingston, NY or Raleigh, NC, USA) and younger female and male C57BL/6J mice were obtained from Jackson Laboratories (JAX, Bar Harbor, ME, USA). All mice were acclimated for at least 2 weeks in our animal facility prior to behavioral testing. The 3–4 month-old young adult mice are referred as the “young” group within the paper. Young and old mouse groups were tested at 3–4 and 22–24 months of age, respectively, and were grouped as follows, divided equally by apparatus: young female (*N* = 10 for each apparatus), young male (*N* = 10 for each apparatus), old female (*N* = 13 for each apparatus), and old male (*N* = 14 for each apparatus). Each group was tested with only one of the mazes to avoid the one-trial tolerance problem (Tucker and McCabe, 2017). All mice received a standard diet [Teklad Global Soy Protein-Free (Irradiated) type 2920X, Envigo, Indianapolis, IN, USA] and water *ad libitum*, were group-housed based on how the mice were received from the source institution with up to 5 mice by sex in ventilated cages with access to a small house and tissues for nesting, and were kept on a 12:12 light: dark cycle at 22°C ± 1 and 30–70% humidity. Adequate measures were taken to minimize animal pain and discomfort. Investigation has been conducted in accordance with the ethical standards and according to the Declaration of Helsinki and national and international guidelines and has been approved by the authors’ institutional review board (approval number AN1920-020).

### Behavior experiments

Two different EPM apparatuses (Stoelting Co., Wood Dale, IL, USA; CleverSys Inc., Reston, VA, USA) were used to compare anxiety-like behavior in young and old mice. The Stoelting maze had arms measuring 50 cm in height, 5 cm in width, and 35 cm in length, with a maze wall height of 15 cm, and was 50 cm in height from the floor.^1^ The CleverSys maze had arms measuring 7 cm in width, 30 cm in length, with a wall height of 15 cm, and was 59 cm in height from the floor. The CleverSys maze also had a small rim of about 3–4 mm surrounding the open arms (Figure 1).^2^ Mice were tested in a neutral, quiet environment between 9:00 and 17:00 (light phase) by the same researchers. Mice were acclimated in their home cage for 1 h in the testing room at 22.5–22.8°C and 30–70% humidity prior to testing. Mice were transported to and from the apparatus in a non-transparent plastic container cleaned with 70% ethanol after each use. Each mouse was picked by the tail and placed in the center of the EPM facing the open arm under 314–368 lx illumination and allowed 5 min to explore the apparatus (Neuwirth et al., 2022). After each trial, the apparatus was extensively cleaned with 70% ethanol to eliminate olfactory cues. All tests were recorded and analyzed using the same software (AnyMaze, Stoelting, Wood Dale, IL, USA), and the duration spent, distance traveled, and number of bouts in the open arms were measured. Briefly, five different areas of the maze were specified using the software drawing tool: closed arm #1, closed arm #2, open arm #1, open arm #2, and the center. The sections remained the same for both apparatuses used, but the specific areas had to be adjusted to reflect the dimension of the maze in use. The testing was recorded using a camera fixed above the maze and the entries in each of the five areas, the time spent as well as the distance traveled were measured using the tracking system set to recognize the nose of the mouse. During the test, the researcher watched the animal perform on the computer screen in real-time to ensure that the tracking was working properly.

### Statistical analysis

Data are presented to three significant digits as mean value (*M*) or percent changes with SEM or confidence interval (CI) and are indicated in the figure legend together with sample size (*N*). Statistical analyses, unpaired *t*-test with Welch’s correction, or two- and three-way ANOVA with either Šídák’s or Tukey *post hoc* multiple comparisons were performed with an α level of 0.05 using appropriate software (GraphPad Prism v. 9, San Diego, CA, USA). Significances are denoted in figures with *p < 0.05, ^**^ *p* < 0.01, ^***^ *p* < 0.001, and ^****^ *p* < 0.0001. Complete analysis for each comparison is reported in the Supplementary material.

## Results

To test whether EPM apparatus construction can affect how old mice perform in the assay as compared to young mice, we obtained two different apparatuses from Stoelting and CleverSys (Figure 1A) and tested young adult (3–4 months) and old (22–24 months) C57BL/6J mice of both sexes for anxiety-like behavior. The differences between the two apparatuses were in color, material, and, in particular, arm width as summarized in Figure 1B. The Stoelting apparatus had an arm width of 5 cm for a total surface area of 175 cm^2^, while the CleverSys apparatus had an arm width of 7 cm for a total surface area of 210 cm^2^. Notably, the open arms of the CleverSys apparatus had a small rim around it of about 3–4 mm, which is not present in the Stoelting maze. Results collected from young and old mice were first analyzed considering sex and apparatus as variables. If no sex differences were found, both sexes were combined for each apparatus to examine EPM performance independent of sex, and if no apparatus differences were found, data obtained from both apparatuses were further combined to determine the overall age-effect on anxiety-like behavior in mice, as measured by the EPM test.

### Distance traveled

**FIGURE 2:**
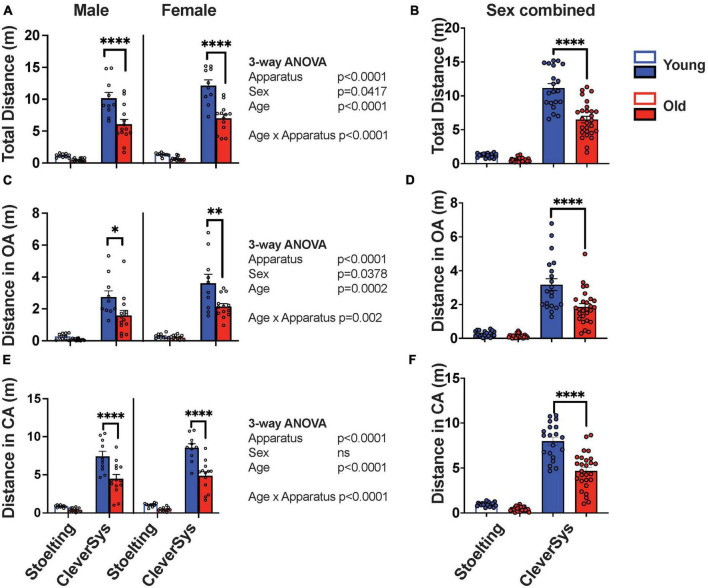
Distance traveled during EPM test. **(A)** Total distance traveled by young and old mice considering sex and apparatus as variables. **(B)** Total distance moved by young and old mice tested with either the Stoelting or CleverSys apparatus independent of sex. **(C)** Distance traveled in the open arms (OA) by young and old mice considering sex and apparatus as variables. **(D)** Total distance moved in OA by young and old mice tested with either the Stoelting or CleverSys apparatus independent of sex. **(E)** Distance traveled in the closed arms (CA) by young and old mice considering sex and apparatus as variables. **(F)** Total distance moved in CA by young and old mice tested with either the Stoelting or CleverSys apparatus independent of sex. Young male Stoelting: *N* = 10, old male Stoelting: *N* = 14, young male CleverSys: *N* = 10, old male CleverSys: *N* = 14, young female Stoelting: *N* = 10, old female Stoelting: *N* = 13, young female CleverSys: *N* = 10, and old female CleverSys: *N* = 13. Data are presented as mean value ± SEM and analyses were performed using either three-way or two-way ANOVA with Tukey or Šídák’s *post hoc* multiple comparisons. Significant levels are indicated respectively: *p < 0.05, **p < 0.01, ^*⁣*⁣**^p < 0.0001.

Since old mice move less when performing behavioral tests, we first analyzed whether age and apparatus construction might affect distance traveled during the EPM assay. Interestingly, we found that all variables tested, including age, sex, and apparatus construction, affected how much mice moved during the EPM test, with age and apparatus construction being the most important factors in determining the distance traveled during the assay [age: *F*(1,86) = 44.00, *p* < 0.0001 and apparatus: *F*(1,86) = 397.4, *p* < 0.0001] (Figure 2A). Old mice moved ∼50% less as compared to young mice, independent of sex and apparatus used for testing; however, Šídák’s multiple comparisons test found that only the mean value of distance traveled by old and young mice tested with the CleverSys apparatus to be significantly different (*p* < 0.0001, 95% CI = [3.350, 5.588]) (Figures 2A, B). Notably, when we compared the total distance traveled by mice tested with different mazes, we found an overall 90% decrease in distance traveled in the Stoelting as compared to the CleverSys maze, which affected both sexes equally and suggests that apparatus dimension is a strong determinant of total movement during the assay [*F*(1,90) = 383.5, *p* < 0.0001] (Figures 2A, B). Further analyses of distance traveled in different maze compartments showed that old mice move less in both open (OA) and closed arms (CA), as compared to younger mice, independent of sex and apparatus [*F*_*OA*_(1,86) = 359.4, *p* < 0.0001; *F*_*CA*_(1,86) = 157.0, *p* < 0.0001] (Figures 2C, E); however, this difference was significant only for mice tested with the CleverSys apparatus (Šídák’s multiple comparisons test open arms: *p* < 0.0001, 95% CI = [0.7179, 1.931] and closed arms: *p* < 0.0001, 95% CI = [2.182, 4.436]) (Figures 2D, F). We also found that females traveled significantly more than males when considering both total distance [*F*(1,86) = 4.275, *p* = 0.0417] and open arms [*F*(1,86) = 4.450, *p* = 0.0378] (Figures 2A, C).

These data thus suggest that characteristics of the apparatus are a strong determinant of distance traveled by mice during the EPM test and key in revealing significant differences between old and young mice.

### Entries in open and closed arms

**FIGURE 3:**
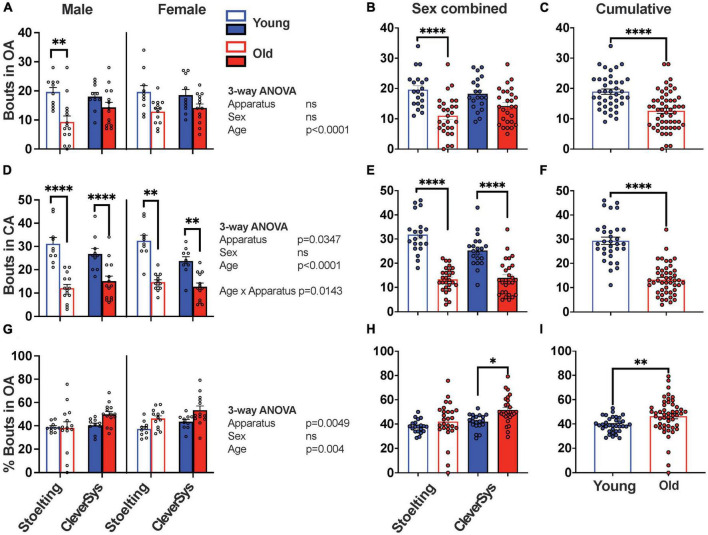
Entries in arms during EPM test. **(A)** Total entries in the open arms (OA) for young and old mice considering sex and apparatus as variables. **(B)** Entries in OA for young and old mice tested with either the Stoelting or CleverSys apparatus independent of sex. **(C)** Entries in OA made by young and old mice independent of sex and apparatus. **(D)** Total entries in the closed arms (CA) for young and old mice considering sex and apparatus as variables. **(E)** Entries in CA for young and old mice tested with either the Stoelting or CleverSys apparatus independent of sex. **(F)** Entries in CA made by young and old mice independent of sex and apparatus. **(G)** Percentage entries in OA for young and old mice considering sex and apparatus as variables. **(H)** Percentage entries in OA for young and old mice tested with either the Stoelting or CleverSys apparatus independent of sex. **(I)** Percentage entries in OA made by young and old mice independent of sex and apparatus. Young male Stoelting: *N* = 10, old male Stoelting: *N* = 14, young male CleverSys: *N* = 10, old male CleverSys: *N* = 14, young female Stoelting: *N* = 10, old female Stoelting: *N* = 13, young female CleverSys: *N* = 10, and old female CleverSys: *N* = 13. Data are presented as mean value ± SEM and analyses were performed using either three-way or two-way ANOVA with Tukey or Šídák’s *post hoc* multiple comparisons or *t*-test with Welch’s correction. Significant levels are indicated respectively: *p < 0.05, **p < 0.01, ^*⁣*⁣**^p < 0.0001.

Next, we analyzed the number of transitions between open and closed arms that young and old mice made during the assay when tested with the two different mazes. When considering entries in the open arms we found that both apparatus construction and sex had no effect, while age significantly affected the OA entries, with old mice transitioning less into the open arms [*F*(1,86) = 25.30, *p* < 0.0001] (Figure 3A). When sexes were combined for each apparatus, Šídák’s multiple comparisons test found that only old mice tested with the Stoelting maze had significantly fewer entries in OA, as compared to young mice (*p* < 0.0001, 95% CI = [3.877, 13.32]) (Figure 3B). Combining data obtained for both apparatuses clearly shows that old mice ventured significantly less in the open arms during the EPM test (unpaired *t*-test with Welch’s correction: *t* = 5.122, df = 88.73, *p* < 0.0001) (Figure 3C).

When measuring entries in the closed arms, we found that apparatus and age significantly affected the number of CA entries with old mice having fewer entries as compared to young mice independent of sex [*F*_*apparatus*_(1,86) = 4.604, *p* = 0.0347; *F*_*age*_(1,86) = 112.0, *p* < 0.0001] (Figures 3D, E). Cumulative data obtained with both apparatuses clearly demonstrates that old mice ventured significantly fewer times in and out of the closed arms during the EPM test (unpaired *t*-test with Welch’s correction: *t* = 9.073, df = 54.05, *p* < 0.0001) (Figure 3F).

Since old mice moved less and had fewer transitions between the open and closed arms as compared to young mice, we proceeded to normalize the data to account for this factor, as previously done in other studies (Shoji et al., 2016; Knight et al., 2021). By expressing the number of entries in the open arms as a percentage of total entries, we found that old mice had a higher percentage of OA entries as compared to young mice [*F*_*age*_(1,86) = 8.773, *p* = 0.0040) (Figure 3G); however, when combining animal sex our results showed that only old mice tested with the CleverSys had a significant increase in percentage of OA transitions as compared to young mice (*p* = 0.0121, 95% CI = [−17.11, −1.886]) (Figure 3H). Combining data from both apparatuses and sexes demonstrated that old mice have a higher percentage of bouts in the open arms as compared to young mice (old vs. young: *t* = 2.983, df = 68.58, *p* = 0.0039) suggesting an overall decrease in anxiety-like behavior with aging as tested using the EPM test (Figure 3I).

### Time spent in arms

**FIGURE 4:**
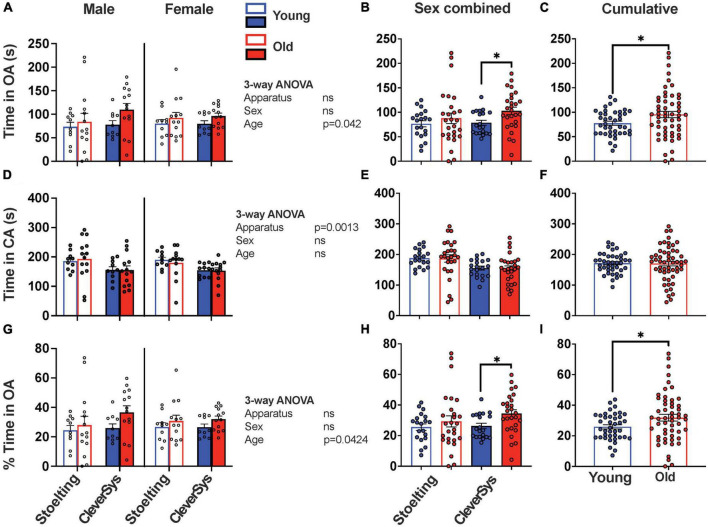
Time spent in open and closed arms during EPM test. **(A)** Time spent in the open arms (OA) for young and old mice considering sex and apparatus as variables. **(B)** Time in OA for young and old mice tested with either the Stoelting or CleverSys apparatus independent of sex. **(C)** Time in OA spent by young and old mice independent of sex and apparatus. **(D)** Total time spent in the closed arms (CA) for young and old mice considering sex and apparatus as variables. **(E)** Time in CA for young and old mice tested with either the Stoelting or CleverSys apparatus independent of sex. **(F)** Time spent in CA by young and old mice independent of sex and apparatus. **(G)** Percentage time spent in OA for young and old mice considering sex and apparatus as variables. **(H)** Percentage time in OA for young and old mice tested with either the Stoelting or CleverSys apparatus independent of sex. **(I)** Percentage time in OA spent by young and old mice independent of sex and apparatus. Young male Stoelting: *N* = 10, old male Stoelting: *N* = 14, young male CleverSys: *N* = 10, old male CleverSys: *N* = 14, young female Stoelting: *N* = 10, old female Stoelting: *N* = 13, young female CleverSys: *N* = 10, and old female CleverSys: *N* = 13. Data are presented as mean value ± SEM and analyses were performed using either three-way or two-way ANOVA with Tukey or Šídák’s *post hoc* multiple comparisons or *t*-test with Welch’s correction. Significant levels are indicated respectively: *p < 0.05.

Lastly, we evaluated the time that the mice spent in the open and closed arms and found that old mice spent more time in OA, as compared to young mice, independent of apparatus and sex [*F*_*age*_(1,86) = 4.262, *p* = 0.0420) (Figures 4A, C); however, Šídák’s multiple comparisons test found that only the mean value of time spent in OA by old and young mice tested with the CleverSys apparatus to be significantly different (*p* = 0.0446, 95% CI = [−48.27, −0.602]) (Figure 4B). When considering the time spent by old and young mice in CA, we found that apparatus but not sex and age affected this variable, with mice tested using the CleverSys maze spending significantly less time in CA as compared to those tested using the Stoelting apparatus [*F*(1,86) = 11.08, *p* = 0.00013] (Figures 4D-F). When time spent in OA was expressed as a percentage of the total time spent in both open and closed arms, we found that old mice had a higher percentage of time spent in OA as compared to young mice (unpaired *t*-test with Welch’s correction: *t* = 2.285, df = 84.67, *p* = 0.0248); however, this difference was statistically significant only for mice tested using the CleverSys apparatus [*F*(1,90) = 4.412, *p* = 0.0385] (Figures 4G-I).

## Discussion

In this study, we analyzed the effect of apparatus construction on anxiety-like behavior in mice assessed by the EPM. We tested the behavior of young adult (3–4 months) and old (22–24 months) C57BL/6J mice of both sexes on two apparatuses (Stoelting and CleverSys) of different dimensions and materials, with one of the major differences being the arm width. Parameters such as distance traveled, number of entries, and duration in the open and closed arms were first evaluated considering apparatus, sex, and age as variables, and then when results obtained from the two apparatuses and different sex were shown to not be significantly different, data were combined to determine the overall effect of age on anxiety-like behavior in mice as assessed by the EPM test.

We found that old mice of both sexes traveled significantly less as compared to young controls in all compartments of the EPM apparatus, including the open and closed arms independent of apparatus used for the test. This is in line with what has been reported in previous studies, which have extensively shown that old mice move less when performing behavioral assays, such as open-field, light/dark transition, and EPM (Shoji et al., 2016; Shoji and Miyakawa, 2019; Tran et al., 2022). We also found, however, that apparatus construction had a dramatic effect on distance traveled by the mice during the test. Notably, both young and old mice were less prone to move in the Stoelting maze, which has narrower arms and lacks a rim around the open arms, as compared to the CleverSys apparatus. Indeed, mice were more prone to fall from the narrow-armed apparatus, which forced the researcher to pause the test and intervene in order to relocate the mouse back on the apparatus. This behavior happened more frequently with older and larger-sized mice, and we cannot exclude that the lack of a small rim around the open arms of the Stoelting apparatus contributed to this effect. Although both apparatuses were able to detect a difference between distance traveled in both open and closed arms by young and old mice, only the data obtained using the CleverSys were significantly different, thus suggesting that apparatus construction is key in highlighting such differences (Figure 2). When we analyzed the number of entries in the open and closed arms, we found that old mice had in general fewer transitions as compared to young mice independent of arm enclosure type (Figures 3A-F), in agreement with other studies (Li et al., 2020). Since the number of transitions in the open arms is an important indicator of anxiety-like behavior in mice, we strongly encourage researchers to report this variable also as percentage of the total number of transitions when working with old animals in order to account for the fewer total transitions, as has been previously done in other studies (Shoji et al., 2016; Knight et al., 2021). Using this normalization step during the analysis revealed that old mice do in fact have a higher percentage of entries in the open arms, indicative of a lower level of anxiety-like behavior (Figure 3I), which was also confirmed by the total and percentage time spent in the open arms (Figures 4C, I). In this regard, however, we also found that apparatus construction was key in revealing significant differences in both percent entries and duration in the open arms between young and old mice (Figures 3H, 4B, H).

Since introduction of the EPM as a test to assess anxiety-like behavior in rodents, numerous studies have been performed to determine factors that could affect outcomes, such as variations in room illumination (Violle et al., 2009), size and color of the apparatus (Lamberty and Gower, 1996; Hagenbuch et al., 2006), as well as exposure to stressors (Wei et al., 2010; Bondar et al., 2018; He et al., 2020). In particular, the effect of apparatus dimensions has been shown to be strain dependent, with C57/BL6 mice more affected than NMRI mice (Lamberty and Gower, 1996), and this has prompted us to investigate whether young and old mice as well as male and female mice might respond differently to changes in EPM apparatus construction. Our findings clearly show that apparatus construction is key in revealing differences in anxiety-like behavior when comparing young and old mice.

While this study successfully addresses the role of apparatus construction in testing old mice for anxiety-like behavior using the EPM assay, it has some limitations that should be discussed. First, the two apparatuses used in this study differed in arm width, material, color, and presence of an edge surrounding the open arms, thus further studies are needed to isolate which of these factors might affect the performance of the mice the most (Shoji and Miyakawa, 2021). A second limitation of our study is that the old and young mice tested were obtained from different facilities, Charles River and Jackson Laboratories, respectively, which limits the possibility to control for exposures of stressors during development and early life that have been shown to affect anxiety-like behavior later on life (Wei et al., 2010; Bondar et al., 2018; He et al., 2020). However, this limitation is only when comparing young and old mice but not when comparing mice within the same age group that are tested on the different apparatuses, i.e., young mice tested on Stoelting vs. young mice tested on CleverSys. Another factor that was not taken into consideration in this study was the estrus cycle of the young female mice during testing. Since the estrus cycle has been shown to cease between 13 and 16 weeks of age, it is not an important variable for old female mice, but it could have an effect in the younger group (Nelson et al., 1982). However, several reports have found no difference in EPM and open-field behavioral performance in C57 mice during different estrus cycle stages (Meziane et al., 2007; Bath et al., 2012; Chari et al., 2020). Lastly, we recognize that in our study we did not do a posture analysis or risk assessment behaviors, which are other common parameters analyzed when conducting the EPM test that could have helped to better understand how the two apparatuses affected mouse performance (Holly et al., 2016).

Taken together, the findings presented in this study support previous studies showing that anxiety-like behavior in mice decreased with age and that EPM apparatus dimensions play an important role in the outcome of the assay and must be considered when testing for anxiety-like behavior in mice for consistency and reproducibility of studies. Our data, as summarized in Table 1, demonstrate that the construction and dimensions of the EPM apparatus have the greatest impact on the total distance traveled in mice independent of animal sex and age. When animal sex and age are also investigated, maze construction also surprisingly affected parameters that measure movements in the open arms, specifically distance traveled, percent entries, duration, as well as percent duration. Furthermore, when data were normalized taking into consideration the fact that older mice move generally less than younger mice, our results clearly demonstrate that old mice show less anxiety-like behavior, as compared to young mice, shown by the higher percentage of entries and time spent in the open arms. These findings, however, were only significant when using the CleverSys maze. Our results, thus, reveal that EPM characteristics affect testing outcomes, with a wider-armed apparatus that has a rim around the open arms to be better suited for studying age-dependent changes in anxiety-like behavior. Lastly, this study suggests that the choice of apparatus should be taken in consideration also when testing pharmaceuticals for their anxiolytic effect since the use of a more anxiogenic apparatus could mask the drug-effect.

**TABLE 1:** Summary of the main parameters affected by the apparatus during the EPM test.

Parameter	Stoelting	CleverSys	Main effect	Variable
Total distance	↓*NS*	↓**S**	↓**S**	Age
	*NS*	*NS*	↓**S^1^**	Sex
Distance OA	↓*NS*	↓**S**	↓**S**	Age
	*NS*	*NS*	↓**S^1^**	Sex
% Entries OA	↑*NS*	↑**S**	↑**S**	Age
	*NS*	*NS*	*NS*	Sex
Time OA	↑*NS*	↑**S**	↑**S**	Age
	*NS*	*NS*	*NS*	Sex

NS, non-significant; **S**, significant; ↓, decrease; ↑, increase.

^1^Females move more than males.

## Data availability statement

The raw data supporting the conclusions of this article will be made available by the authors, without undue reservation.

## Ethics statement

The animal study was reviewed and approved by The University of Rhode Island Institutional Animal Care and Use Committee.

## Author contributions

LG, SB, GC, and JR conceived the study, designed the experiments, and wrote the manuscript. LG and SB performed the experiments and analyzed the data. All authors had read and approved the final manuscript.

## Footnotes

1
https://stoeltingco.com/Neuroscience/Elevated-Plus-Maze~9853

2
https://cleversysinc.com/CleverSysInc/csi_products/e-p-maze/
